# Control Group Design: Enhancing Rigor in Research of Mind-Body Therapies for Depression

**DOI:** 10.1155/2013/140467

**Published:** 2013-04-07

**Authors:** Patricia Anne Kinser, Jo Lynne Robins

**Affiliations:** Virginia Commonwealth University, School of Nursing, 1100 East Leigh Street, Richmond, VA 23298, USA

## Abstract

Although a growing body of research suggests that mind-body therapies may be appropriate to integrate into the treatment of depression, studies consistently lack methodological sophistication particularly in the area of control groups. In order to better understand the relationship between control group selection and methodological rigor, we provide a brief review of the literature on control group design in yoga and tai chi studies for depression, and we discuss challenges we have faced in the design of control groups for our recent clinical trials of these mind-body complementary therapies for women with depression. To address the multiple challenges of research about mind-body therapies, we suggest that researchers should consider 4 key questions: whether the study design matches the research question; whether the control group addresses performance, expectation, and detection bias; whether the control group is ethical, feasible, and attractive; and whether the control group is designed to adequately control for nonspecific intervention effects. Based on these questions, we provide specific recommendations about control group design with the goal of minimizing bias and maximizing validity in future research.

## 1. Introduction

As the state of the science progresses in the field of complementary and alternative medicine (CAM), it is imperative for researchers to develop well-designed clinical trials that clearly and systematically develop a base of evidence to support effects and efficacy of mind-body therapies. Yoga and tai chi are two mind-body therapies that hold promise as complementary therapeutic interventions for clinical conditions such as depression. It is relevant and important to test these interventions with appropriately designed randomized controlled trials (RCTs). However, numerous methodological issues have been cited in research in this area, such as a lack of consistency between research question and design, unclear treatment protocols and reporting of findings, and lack of appropriate bias controls, among others (for additional perspectives on general methodological challenges with various mind-body modalities, see [1–7]). Although guidelines have been proposed for the development of yoga and tai chi interventions for randomized trials and recommendations have been made about the appropriate reporting of research findings, one important methodological dilemma that has remained largely unexplored in the literature is the issue of appropriate control group design [4, 7, 8].

Because control group design continues to be a challenge for researchers, the purpose of this paper is to provide perspectives on important considerations and make recommendations about control group design in mind-body therapy research. The motivation for this paper arose because of the challenges faced by these authors in the design of control groups for their recent clinical trials of mind-body complementary therapies for women with depression. Examples from related research studies and “lessons learned” from our studies will be provided in order to elucidate some of the more salient issues in control group design. While the majority of the literature cited focuses on two mind-body therapies, yoga and tai chi, the control group dilemmas elucidated here are applicable to other mind-body research.

## 2. Methods and Results

### 2.1. Control Group Defined

It is one of the most basic yet critical aspects of an RCT; control groups are necessary for discriminating treatment outcomes from outcomes related to other factors, such as the natural history of a disease process and participant or researcher expectations. If a participant experiences a clinical improvement during a study, a well-designed study with an appropriate control group will enable the improvements to be attributed to the intervention itself, thus strengthening the validity and credibility of the findings. As such, the design of a control group is as critical as the design of the intervention group.

### 2.2. Control Group Design in Pharmaceutical Research

Although pharmaceutical research designs may be more straightforward than designs for examining multifaceted mind-body interventions, a brief examination of control group design in pharmaceutical research provides relevant context. The Food and Drug Administration (FDA) is clear about the need for “adequate and well-controlled” examinations of the effects of a particular treatment in drug studies [9]. The FDA suggests that there are five types of control groups appropriate for rigorous studies [10]: the concurrent placebo; the no-treatment concurrent control; the dose-response concurrent control; the active/positive concurrent control; and the external/historical control. The FDA states that, although these various types of control groups may be relevant in various contexts, there are certain caveats for each of them. First, placebo-only control groups are not recommended when effective and established treatments exist for ethical reasons. Second, the no-treatment control group is only reasonable when the study outcomes are completely objective and cannot be influenced by the lack of blinding. The dose-response concurrent control is used to compare groups' responses to variable doses of the drug and can be valuable only after initial testing. Fourth, the use of an active control group requires focusing on outcomes that are clinically important because there may not be very large effect sizes or obvious statistical significance of differences in outcomes. Importantly, active control group designs pose fewer ethical dilemmas than placebo controls, particularly when a new treatment is expected to be at least as good as the existing treatment. 

### 2.3. Control Group Design in Mind-Body Research

The design of control groups in clinical trials with nonpharmacological, psychosocial, and/or behavioral interventions is often much more complex than those of drug studies. Researchers investigating mind-body complementary interventions for clinical conditions have a number of challenges in optimal study design, predominantly because these interventions are typically multifaceted and complex (e.g., they often involve multiple components: behavioral, psychosocial, and educational). Many of the control group options listed above are not appropriate for this type of research; for example, the placebo control is often not feasible because mind-body therapies do not typically have an obvious placebo or “sham” intervention. Researchers of mind-body therapies involving psychosocial/behavioral interventions are challenged to identify appropriate psychosocial or behavioral “placebos” because the active/inactive components of interventions are not as apparent as they are for medications [11]. Additionally, a no-treatment control group is often unethical when conducting research with clinical populations (such as individuals with depression) because there is typically a known effective therapy or a minimum level of “usual care” expected depending upon the severity of the clinical condition. 

Given these caveats, the most common types of control group conditions used in research involving mind-body interventions include (1) the “usual care control,” whereby participants receive the usual care for the clinical condition, (2) the “wait-list control,” whereby participants will receive the usual care and will later receive the intervention in addition to the usual care, (3) the “active control,” whereby the control group receives an activity or intervention which controls for some aspect of attention, time, or expectation, and (4) the “dismantling” or “add-on” control, where components of an existing intervention are isolated or added on in an attempt to identify the essential mechanism of action [11, 12]. However, specific challenges are often present with these types of control groups in mind-body research, as seen in the literature and in our experiences with recent RCTs involving yoga and tai chi discussed below. It should be noted that the National Center for Complementary and Alternative Medicine defines mind-body practices as those which promote health by facilitating interactions among the brain, mind, body, and behavior (e.g., meditation, yoga, tai chi, guided imagery, and others) [11]; we focus herein on two specific mind-body therapies, yoga and tai chi, that are receiving increasingly more attention in the research and lay communities for their potential effects in depressed patients [13]. 

#### 2.3.1. Control Groups in Yoga for Depression Studies

A review of RCTs of yoga as a mind-body complementary intervention for depression reveals that the design of control groups is highly varied (Table 1). The most common type of comparison group is the usual care or wait-list control where no changes were made to the typical activities of the participants in those groups [14–16]. Three out of the six studies used some sort of active control. The study by Butler and colleagues used a psychoeducational activity as the active control group in which participants were to read a book and pamphlets provided to them [17]. The attention from study staff was quite different between the two groups, whereby the control group did not involve any group or individual sessions, yet the intervention groups both had 20 hours of interactions with other participants and research staff. The study by Sharma and colleagues was unique in its use of an active control which is not commonly used in yoga research. In this case, a “sham” activity was designed to mimic the movements and stillness of the yoga intervention but details about the components of both the intervention and control group are lacking [18]. Finally, one study compared the use of a specific type of yoga (Laughter Yoga) in Iranian elderly depressed women to two control groups a usual care control and an exercise control group [19]. The exercise group (jogging and stretching) did control for attention and time, by mirroring the number and length of the yoga group sessions.

Although many research studies have sought to examine the efficacy and effectiveness of yoga for depressed individuals, the control group design has been inconsistent and often not thoroughly discussed. Furthermore, it is unclear whether the control groups used in these studies were able to control for “co-intervention” or the unequal attention given to the yoga group [6]. In almost all of these studies, equivalent time and attention were not provided to the control group. As such, one must consider whether the outcomes of interest were affected.

#### 2.3.2. Control Groups in Tai Chi for Depression Studies

A review of RCTs of tai chi as a mind-body complementary therapy for depression also reveals that the design of control groups is varied, including no-treatment control, wait-list control, and active control groups (Table 2). A total of three of the five studies meeting the search criteria employed wait-list control groups and the treatment/wait-list conditions lasted three months [20–22]. In a wait-list control group design, participants assigned to the control group served as the untreated comparison for the treatment group and later received the intervention. The other two studies used active control groups. Lavretsky and colleagues sought to examine the potential benefit of tai chi in partial responders to the drug escitalopram [23]. The active control group involved a health education activity; the authors rationalized this because they wanted to determine if tai chi would have a clinical benefit independent of other treatment factors such as expectation and group support. Cheng and colleagues randomized participants to receive either the tai chi class (first intervention group) or to play a game of mahjong (second intervention group) or to participate in the handicraft group (control group) which consisted of connecting beads together to create interesting shapes [24]. Each activity was performed for one hour three times a week for 12 weeks. This handicraft activity was designed to control for group effect.

This brief, focused review of the literature provides evidence that a variety of control group conditions are being employed in studies of tai chi for depression. While it is useful that the various authors have acknowledged the challenges in selecting an appropriate control group by explaining their choices, there is not a clear answer for the strongest control group design. The wait-list control group design is a reasonable design but does introduce potential bias given the sense of expectancy it creates in the control group. The active control group chosen was well-documented and matched the time and attention given to the tai chi intervention group.

#### 2.3.3. Lessons Learned from a Recent Study on Yoga for Depression

We recently conducted a small RCT to evaluate the feasibility, acceptability, and effects of yoga for women with major depressive disorder (MDD) and residual symptoms despite the usual care [25]. Conducted in a convenience sample of 27 women, this community-based prospective, randomized, and controlled pilot study used a mixed-methods approach in comparing an 8-week gentle Hatha yoga intervention and an active control activity. In the yoga intervention group, participants took part in once-weekly 75-minute group classes for 8 weeks; they were also encouraged to do yoga daily at home using handouts and/or a provided DVD. In the attention-control group, participants engaged in a series of weekly 75-minute health education sessions involving lectures, discussions, and videos for 8 weeks; participants were also encouraged to review the health concepts at home using provided handouts and websites. Both groups had an equivalent numbers of visits (screening, intervention points, data collection) and phone contacts with study staff. 

The design of the active control group seemed reasonable and appropriate for several reasons. Although the majority of the recent RCTs on yoga for depression have used usual care or wait-list controls, health education sessions were chosen as an active control activity because the depression intervention literature suggests that health education sessions are reasonable activities with depressed participants and have been effective in retaining control group participants in this manner [26–32]. Additionally, the health education sessions provided equivalent group class time and exposure to study staff. Both groups were provided with handouts and were equally expected to complete the “homework.” Participants in the attention-control group had equal face-to-face time with other participants, in the attempt to control for the effect of group interactions. To maintain internal validity, the lectures and videos were designed to avoid content overlap with material presented in the yoga intervention. Because the yoga intervention was comprised of both behavioral and psychoeducational components (physical activity, breathing, relaxation, and discussions of incorporation of yogic principles into daily life), the control group activity was designed to exclude this content, such that the control activity involved only psychoeducation about wellness topics. Finally, the active control group involved group discussions and videos in the attempt to be interesting enough to retain participants. Both groups received a small compensation for completion of the study to minimize overall attrition. 

Despite the fact that the control group intervention was designed thoughtfully and purposefully, we experienced a number of challenges with the control group. First, there was a higher attrition rate in the control group, perhaps due to disappointment of not being randomized into the yoga group. Because part of the informed consent process involved informing potential participants about both groups, it was impossible to blind participants to the fact that yoga was a possible intervention. Second, by learning about and discussing healthy behaviors in the weekly sessions, the control group participants ended up making more significant behavior changes than was expected for an 8 week intervention. For example, a number of participants engaged in close social support and started going to the gym and attending group psychotherapy sessions together. The fact that these participants had significant decreases in depression may have been partly related to the health education sessions, but even more likely they were related to the social support and discussions between the women. Third, social support may have been stronger in the health education group because more time was allowed for discussion in class. As such, getting women together on a weekly basis may have become an intervention in and of itself, a subtle form of group therapy, ultimately diluting and confounding the findings.

#### 2.3.4. Lessons Learned from a Clinical Trial of Tai Chi in Women with Breast Cancer

Tai chi was one of two interventions in a longitudinal RCT in women with early stage breast cancer during the period of chemotherapy. A study aim was to compare the effects of tai chi to a spiritual growth group and a no-treatment control that received standard care on psychosocial functioning including depression. Recruitment and retention presented significant challenges in this study in part due to the timing of the intervention as well as the standard care control group. The enrollment of women into a longitudinal study while they were in the process of acclimating to the diagnosis of breast cancer as well as making difficult treatment decisions impacted recruitment and retention. Additionally, although women were intrigued by the opportunity to participate in a study involving tai chi and spiritual growth groups, their interest seemed to wane when assigned to the usual care control group, thus creating further challenges with retention. While a wait-list design was not possible because of the longitudinal study design, an active/attention-control group such as general breast cancer related health education might have helped with recruitment and retention. 

## 3. Discussion: Considerations and Recommendations for Future Research

A growing evidence base indicates that certain mind-body therapies may be appropriate in patients with depression, yet almost every integrated review or meta-analysis finds that the literature lacks methodological rigor including the lack of an appropriate control. Although the double- or triple-blinded RCT has historically been considered the “gold standard” in considering the quality of research, this design does typically not lend itself well to mind-body intervention studies. That said careful design of these studies is critical if we are to be able to evaluate the effectiveness of these interventions in order to facilitate appropriate integration into healthcare. Because of the nature of multifaceted interventions such as yoga and tai chi, it is imperative to design the best control group(s) in order to minimize bias and maximize validity and meet the specific needs of the research (see Table 3 for considerations about types of control groups in mind-body research studies). Furthermore, in order to be consistent with recommendations from the Consolidated Standards of Reporting Trials (CONSORT) Statement, transparent reporting of decision making in control group design is an essential aspect of the conduct of research on mind-body therapies [33, 34].

### 3.1. Key Questions and Recommendations Regarding Control Group Design

Based upon our brief review of the literature, our experiences in designing RCTs on yoga and tai chi for depression, and recommendations from the National Institutes of Health, it is apparent that the researcher must make careful decisions when designing the control group, in order to hold as many factors constant as possible between the intervention and control conditions, in order to illuminate the hypothesized mechanism of action [11, 35]. The following key questions arise regarding appropriate study and control group design:

1. Does the study design match the research question?
2. Does the control group address performance, expectation, and detection bias?
3. Is the control group ethical, feasible, and attractive?
4. Does the control group control for nonspecific aspects of the intervention?

**Figure 1:**
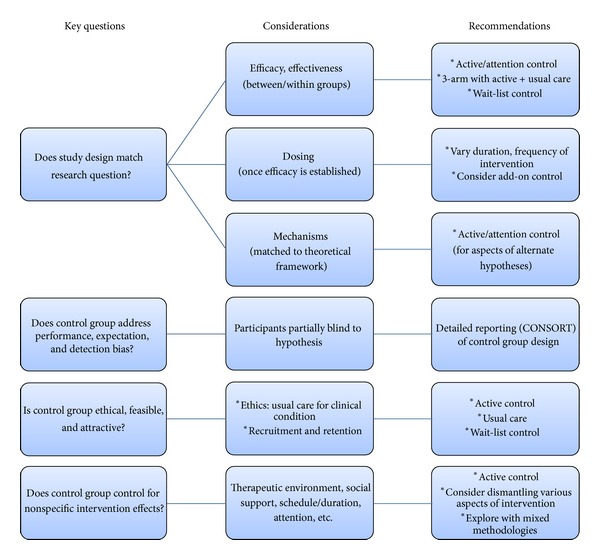
Key questions, considerations, and recommendations about control group design.

Figure 1 provides a visual illustration of these questions along with the important considerations and recommendations discussed herein.

First, in order to enhance the validity and applicability of mind-body therapy research, the choice of the best control group should be determined by the research question. The current efficacy literature has tended toward the use of a usual care or wait-list control group, and these designs are traditionally considered to be somewhat “weak” because there is no control for nonspecific treatment effects and it is more difficult to differentiate the treatment effects [12, 35]. Future researchers should consider the following guidelines regarding the goal of the research and the best study design: (a) if a researcher seeks solely to establish efficacy and effectiveness (i.e., *determine whether and to what degree* a mind-body intervention may be helpful to depressed individuals), the study design should evaluate within subjects and between groups. As such, the suggested control group would be an active control, a wait-list control, or a 3-arm design with an active control group and a usual care control group; (b) If the goal of the research is to determine appropriate dosing of a mind-body intervention then the study may not include a simple usual care group but rather would involve multiple arms with varying duration/frequencies of the intervention among the comparison groups. An “add-on” group or a dismantling study may also be appropriate. However, efficacy of the intervention must be determined prior to a dosing study [9]. (c) If a researcher seeks to determine underlying mechanisms for the effects of these interventions, then the study design must depend upon a theoretical framework with acknowledgement of plausible alternate hypotheses (see full discussion in [1]). In this case, to enhance the rigor of the study, the control group would be designed to actively control for the alternative explanations for the possible effects of the intervention (e.g., group effect/social support, attention from study staff, therapeutic environment, etc.) [36]. 

Second, the scientific community often calls for study design to minimize performance, expectation, detection, and selection bias [37]. However, the method for doing so usually involves double- or triple-blinding and the use of placebo controls, none of which are typically feasible in research on mind-body modalities such as yoga and tai chi. Most often, there are no reasonable options to serve as a placebo or a “sham” intervention to allow for full blinding of participants or researchers [38]. Despite this, researchers may consider creative blinding methods, such as partially blinding participants to the study hypothesis and blinding the data analysts [7, 39]. Finally, researchers may overcome scientific concerns about methodological rigor by providing detailed reports of recruitment, randomization, and data analysis processes using the guidelines from the CONSORT Statement and those by Boutron and colleagues regarding methods to extend the CONSORT Statement to nonpharmacologic treatments [7, 34].

Third, a consideration of the ethics, feasibility, and attractiveness of a control group is always warranted. For example, in research of mind-body complementary therapies for depression, researchers must ask an important question: what is a safe, ethical, and appropriate control group when the clinical population is depressed? No-treatment groups are not ethically reasonable because the usual care for depression has been clinically established in the form of psychoactive medications and psychotherapy. It is ethical to expect that participants continue to receive this usual care when a new intervention is tested. While the use of a simple usual care control group is often the most feasible, this may limit the attractiveness for potential participants. Despite widespread use, the usual care for depression has many limitations including patient reluctance to take medications, lack of access to qualified psychotherapists related to cost or availability, and patients being refractory to treatment, among others [40]. Furthermore, researchers must consider how to control for expectation and how to prevent the high rate of attrition typically seen in control groups, particularly in depressed study populations. Researchers often add elements to the active control group that are attractive to those randomized to the group. The control group activities must be attractive enough to maximize retention while simple enough to prevent significant changes in participants' behavior [41]. Care must be taken to avoid enacting unintentional change in participants in the control group [12]. Active control group activities that deliver some kind of education may end up changing participants' behaviors and thus outcomes, becoming an intervention in and of themselves.

Fourth, researchers must consider whether and how the control group may account for nonspecific aspects of the intervention such as the therapeutic environment, social support, schedule and duration of practice of the treatment and control activities, attention from study staff, and other factors. For example, in research of an intervention for depression, the usual care involves the standard unilateral or bilateral approach of antidepressant drug therapy with or without psychotherapy. In a usual care control group, the participants would receive nothing more than these standards of care. Alternatively, in an active control group, the participants may continue to receive the usual care plus a researcher-designed intervention that controls for some aspects of the attention that the intervention group receives. Ideally, the intervention group and the nonspecific active control group should parallel one another with regard to attention from and contact time with the research staff, time spent in the research-related activities (from group meetings to phone calls or length of involvement in weeks/months), social support, follow-up times, and other similar factors; this allows for any differences in the groups to be attributed to the intervention itself, rather than these factors [12]. The active control design is quite often ideal because it assists in controlling for these nonspecific features of an intervention such as number of visits, timing of the intervention, and time/attention spent with participants. Ultimately, for researchers designing a study of a mind-body complementary intervention for depression, the control group design that may be most reasonable is the active control.

### 3.2. Additional Recommendations for the Future

Along with the recommendations provided above, what else could be done to optimize the design of a control group in future larger-scale studies on mind-body modalities? There are a few additional options that must be evaluated and carefully considered, each with its own benefits and challenges.

Researchers must design a clear, detailed, and replicable intervention which may ultimately simplify the type of control group to use. One method may be the use of a dismantling study, in which components of an intervention are broken down and evaluated individually. One benefit of these types of studies is that isolating individual components of an intervention may allow for the intervention to be kept as simple as possible and the optimum control activity may become clear. For example, real-world mind-body interventions often involve a multitude of potentially beneficial factors: gentle physical movement, relaxation, breathing, mindfulness, calming music in the background, and guided imagery, among others; it may be difficult to determine whether one should control for these aspects. In a dismantling study, the choices become more clear as a researcher chooses to focus only on, say, the physical movement of yoga postures, and therefore the control group would have to involve another type of exercise (see Streeter and colleagues' study comparing yoga asana to a walking control group) [42]. This could also be helpful for evaluating the cost-effectiveness of an intervention such as yoga for depression. We acknowledge that this is often not the real-world experience of these mind-body therapies and that this could significantly decrease the effect size of an outcome, yet a dismantling study may be reasonable in some cases to allow for close examination of the effects of certain components of the intervention and to potentially enhance methodological rigor. 

Researchers may also consider multiarm studies in which various types of control and intervention groups are used, particularly for considering comparative effectiveness. Instead of dismantling potentially important components of an intervention, as discussed in the previous paragraph, the researcher may dismantle the location of the intervention and control groups. For example, our intervention involved both home and group classes which were time-intensive and involved travel. Although we did find that participants reported enjoying the group classes more than home practice, there was no way to compare whether one has a greater effect than the other on depressive symptoms. A four-group design could be implemented that dismantles the location of yoga, with groups as follows: (1) usual care for depression, (2) usual care + home yoga practice, (3) usual care + group yoga class, and (4) usual care + group and home yoga practice. This may assist, also, in controlling for the social support that often inadvertently occurs in any group setting. The risk with this type of dismantling may be that the similar interventions could result in smaller effect sizes; additionally, the overall sample size must be large enough to prevent a Type 2 error.

In addition, we highly recommend that researchers use mixed methodologies in research on mind-body interventions, in which both qualitative and quantitative approaches are integrated in the study design. This is beneficial because qualitative data may provide a more in-depth perspective on participants' experiences in both the intervention and control activities, as we experienced in our recent clinical trials. For example, participants may reveal in an interview whether and how social interactions occurred within a treatment or control group or may discuss individual variations in life stresses and social resources. This qualitative data may greatly enrich a study's quantitative findings from psychological and physiological objective data. Despite the unique challenges presented in the conduct of qualitative research (e.g., bracketing research biases, among others; for more in-depth discussions, see [43–46], among others), the benefits outweigh the challenges and should be considered for the value added to research of mind-body interventions.

As mentioned previously, in order to minimize bias and enhance the validity of a study, researchers should consider creative methods for blinding with regard to treatment allocation and the study hypothesis. Inadequate concealment of allocation and hypothesis may cause an exaggeration of treatment effects due to participant and researcher expectations [39, 47, 48]. Furthermore, inadequate concealment of the study hypothesis may lead to high rates of attrition in a control group, whereby the control group participants are less motivated to stay in a study if they perceive that a “new, promising” intervention is more efficacious than the standard treatment they are receiving [39]. To prevent these issues, partial blinding of participants to the study hypothesis may enhance retention and lend validity to study findings. In addition, investigators and study staff should be blinded as much as possible when conducting qualitative interviews and data analysis in order to prevent observer bias.

Finally, researchers must be as transparent as possible in their reporting of decision-making steps in study design, guided by the CONSORT Statement and related revisions [7, 33, 34]. Publications of research findings must include any assumptions or decision points about the critical components or unique aspects of an intervention and the control activity. For example, it is important to acknowledge that there may be additive effects of a yoga or tai chi intervention based on the environment (pleasing appearance of room or music playing), the personality, and skill set of study staff/instructors, among others. For example, in designing an appropriate control group for our yoga study, we clearly designed the health education sessions to mirror the time, location, and study staff attention as received in the yoga group. Enhanced transparency of the conduct of a clinical trial is necessary for other researchers to be able to evaluate sources of bias, understand how an intervention was actually administered, and even conduct meta-analyses [47]. For the purpose of advancing the science and translating research into clinical practice as well as making these therapies more available to populations that may benefit from them, the goal of well-designed research is to deliver valid conclusions. Clear descriptions of interventions and control groups may be one effective step to do so.

## 4. Conclusion

In conclusion, the state of the science in mind-body research is that every systematic review has identified the need for better methodology and more well-controlled studies. Considering the challenge in developing optimal controls, we began with a brief focused review of control groups used in pharmaceutical research. Given that the pharmaceutical sector's gold standard of randomized and double-blinded design does not typically apply in mind-body therapy research, we briefly reviewed the types of control groups used in the literature on yoga and tai chi for depression. To address the methodological challenges of research on mind-body modalities, we have reviewed key questions researchers should ask when designing control groups and we have suggested multiple ways to minimize bias and maximize validity. We have focused our discussion and recommendations based upon research of two types of mind-body interventions, yoga and tai chi, for depression. However, the recommendations here may be appropriately applied in other modalities and conditions typically examined in complementary and alternative therapy research. Although we acknowledge that these methods are not the only ways for minimizing complications, we expect that this paper begins the discussion amongst our research colleagues about these and other methods for optimal research study design.

## Figures and Tables

**Table 1:** Comparison of intervention and control group conditions in community-based RCTs of yoga for adults with depression.

Author, date	Intervention group(s)	Control group(s)
Woolery et al. (2004) [16]	Group class: Iyengar yoga 1 hour twice per week × 5 weeks	Wait-list control

Sharma et al. (2006) [18]	Home practice after initial group training: Sahaja yoga (meditation with specific hand movements) 30 minutes three times per week × 8 weeks	Placebo control: sham meditation control (hands in different positions, sitting with eyes closed) 30 minutes three times per week × 8 weeks

Butler et al. (2008) [17]	(a) Group class: Hatha yoga + psychoeducation 2 hours per week × 8 weeks + plus 4-hour booster session at week 12; home practice: 30 minutes daily 6 days per week recommended with manual and tapes (b) Group class: hypnosis + psycho-education 90 minutes per week × 10 weeks plus 4-hour booster at week 12	Active control: psycho-education alone (reading materials—no group sessions)

Krishnamurthy and Telles (2007) [15]	(a) Group class: yoga 30 minutes per week × 24 weeks (b) Ayurveda herbal therapies	Wait-list control

Field et al. (2012) [14]	(a) Group class: yoga 20 minutes per week × 12 weeks (b) Individual massages 20 minutes per week × 12 weeks (delivered by researchers)	Usual care control

Shahidi et al. (2011) [19]	Group Laughter yoga 30 minutes × 10 sessions	(a) Active control: group exercise 30 minutes × 10 sessions (b) Usual care control

The literature review inclusion criteria: community-based, randomized, and controlled studies published in 2000–2012; English; participants with confirmed diagnosis of depression/dysthymia or high levels of depressive symptoms; yoga intervention group. Exclusion criteria: participants who were inpatient/hospital-based; had no clinical or confirmatory diagnosis of depression/dysthymia; nonrandomized and noncontrolled designs; participants were excluded if they reported a psychiatric diagnosis.

**Table 2:** Comparison of intervention and control group conditions in community-based RCTs of tai chi for adults with depression.

Author, date	Intervention group(s)	Control group(s)
Chou et al. (2004) [20]	45 minute tai chi class 3 times weekly for 3 months	Wait-list control
Cho (2008) [21]	3 tai chi classes per week for 3 months	Wait-list control
Lavretsky et al. (2011) [23]	2-hour tai chi class weekly for 10 weeks	Active control: health education attention-control 2-hour class weekly for 10 weeks
Cheng et al. (2012) [24]	1-hour tai chi class or mahjong game 3 times weekly for 3 months	Active control: handicrafts
Yeung et al. (2012) [22]	1-hour class twice weekly for 12 weeks	Wait-list control

The literature review inclusion criteria: community-based, randomized, and controlled studies published in 2000–2012; English; participants with confirmed diagnosis of depression; tai chi intervention group. Exclusion criteria: participants were inpatient/hospital-based; no clinical or confirmatory diagnosis of depression; non-randomized and non-controlled design; participants were excluded if they reported a psychiatric diagnosis.

**Table 3:** Considerations about types of control groups in studies of mind-body therapies.

Control groups	Pros	Cons
Usual care control	(i) Ethical	(i) Variability in usual care for depression according to the individual (ii) Attrition

Wait-list control	(i) Ethical (ii) Enhance recruitment and retention	(i) Expectation bias (ii) Does not allow for blinding of participants to hypothesis (iii) Extends study time (iv) Waiting too long for intervention may lead to attrition (v) No control for non-specific treatment effects

Active control	(i) Ethical (ii) Enhance recruitment and retention (iii) May allow for blinding of participants to hypothesis (iv) Control for threats to internal validity	(i) Must exactly parallel treatment group in time and attention (ii) May become an intervention in and of itself (iii) May be more difficult to detect treatment effect
